# Keywords reflecting sepsis presentation based on mode of emergency department arrival: a retrospective cross-sectional study

**DOI:** 10.1186/s12245-021-00396-z

**Published:** 2021-12-20

**Authors:** Ulrika Margareta Wallgren, Eric Larsson, Anna Su, Jennifer Short, Hans Järnbert-Pettersson, Lisa Kurland

**Affiliations:** 1 Department of Clinical Science and Education, Karolinska Institutet, Söderssjukhuset, Sjukhusbacken 10, 118 83 Stockholm, Sweden; 2 Fisksätra Vårdcentral (Primary Health Care Center), Fisksätra torg 20, 133 41 Saltsjöbaden, Sweden; 3 Department of Medical Sciences, Örebro University, Campus USÖ, Södra Grev Rosengatan 32, 701 12 Örebro, Sweden; 4 Department of Surgery, Sankt Göran Hospital, Sankt Göransplan 1, 112 19 Stockholm, Sweden; 5 Department of Urology, Linköping University Hospital, 581 85 Linköping, Sweden; 6 Department of Emergency Medicine, Örebro University Hospital, Södra Grev Rosengatan 18, 703 62 Örebro, Sweden

**Keywords:** Emergency medical service, Emergency care, Emergency department, Sepsis, Symptoms

## Abstract

**Background:**

Current sepsis screening tools are predominantly based on vital signs. However, patients with serious infections frequently present with normal vital signs and there has been an increased interest to include other variables such as symptoms in screening tools to detect sepsis. The majority of patients with sepsis arrive to the emergency department by emergency medical services. Our hypothesis was that the presentation of sepsis, including symptoms, may differ between patients arriving to the emergency department by emergency medical services and patients arriving by other means. This information is of interest to adapt future sepsis screening tools to the population in which they will be implemented. The aim of the current study was to compare the prevalence of keywords reflecting the clinical presentation of sepsis based on mode of arrival among septic patients presenting to the emergency department.

**Methods:**

Retrospective cross-sectional study of 479 adult septic patients. Keywords reflecting sepsis presentation upon emergency department arrival were quantified and analyzed based on mode of arrival, i.e., by emergency medical services or by other means. We adjusted for multiple comparisons by applying Bonferroni-adjusted significance levels for all comparisons. Adjustments for age, gender, and sepsis severity were performed by stratification. All patients were admitted to the emergency department of Södersjukhuset, Stockholm, and discharged with an ICD-10 code compatible with sepsis between January 1, and December 31, 2013.

**Results:**

“Abnormal breathing” (51.8% vs 20.5%, *p* value < 0.001), “abnormal circulation” (38.4% vs 21.3%, *p* value < 0.001), “acute altered mental status” (31.1% vs 13.1%, *p* value < 0.001), and “decreased mobility” (26.1% vs 10.7%, *p* value < 0.001) were more common among patients arriving by emergency medical services, while “pain” (71.3% vs 40.1%, *p* value < 0.001) and “risk factors for sepsis” (50.8% vs 30.8%, *p* value < 0.001) were more common among patients arriving by other means.

**Conclusions:**

The distribution of most keywords related to sepsis presentation was similar irrespective of mode of arrival; however, some differences were present. This information may be useful in clinical decision tools or sepsis screening tools.

**Supplementary Information:**

The online version contains supplementary material available at 10.1186/s12245-021-00396-z.

## Background

Sepsis, defined as life-threatening organ dysfunction caused by a dysregulated host response to infection [1], is one of the most common medical emergencies and affects approximately 19 million people worldwide each year [2]. Septic patients are in need of urgent medical care and early treatment requires early identification. However, sepsis is often difficult to identify due to a wide variety of presenting symptoms [3, 4].

Most screening tools designed to identify sepsis within emergency care are based on vital signs alone [5, 6] despite the fact that one-third of all patients with severe infection present with normal vital signs [7]. Therefore, one may conclude that variables other than vital signs need to be considered in addition to vital signs in order to increase the identification of the septic patient.

Keywords related to septic patients’ symptom presentation have previously been shown to follow a pattern among septic patients presenting to the emergency medical services (EMS), [3]. However, septic patients arriving to the emergency department (ED) by EMS have previously been shown to present with a more severe condition than patients arriving by other means [8]. Hence, we are interested in understanding whether the presentation of sepsis differs depending on mode of ED arrival.

The aim of the current study was to compare the prevalence of keywords reflecting the clinical presentation and mode of arrivalamong septic patients presenting to the emergency department

## Methods

### Study design and setting

This was a retrospective cross-sectional study of 479 adult patients admitted to the ED of Södersjukhuset between January 1, and December 31, 2013, and discharged with an International Classification of Disease, tenth revision (ICD-10) code compatible with sepsis.

The ED of Södersjukhuset is an urban 603-bed teaching hospital in Stockholm, Sweden, with more than 129,000 adult ED visits annually [9].

### Selection of study participants

#### Inclusion criteria

Adult patients (≥ 18 years of age) admitted from the ED to in-hospital care at Södersjukhuset that upon discharge from in-hospital care received an ICD-10 code (primary and/or secondary diagnosis) compatible with sepsis (A02.1, A22.7, A26.7, A32.7, A39.2, A39.4, A40.0-A40.3, A48-A49, A41.0-A41.5, A41.8-A41.9, A42.7, B37.7, R57.2, R65.0-65.1) were included. Also, patients that died during in-hospital care were included in the current study.

#### Exclusion criteria

Exclusion criteria were (1) healthcare-associated infections (HCAI), defined as sepsis with an onset ≥ 48 h after admission to the ED [10], (2) EMS transport of patients already being treated for sepsis or an infection, (3) unknown mode of arrival, and (4) lack of ED and/or EMS record.

### Definitions

Sepsis was defined as discharge from in-hospital care with an ICD-10 code compatible with sepsis (see the “Inclusion criteria” section above). The study material was collected during the period when the Sepsis-2 criteria [11] were in use.

Severe sepsis was defined, in accordance to a previously developed definition adapted to emergency care [12], see Additional file 1.

The EMS group was defined as patients arriving to the ED by EMS, i.e., ambulance or ambulance helicopter. The non-EMS group was defined as patients arriving to the ED by any means of transportation other than EMS, i.e., walking, by private car/taxi, or police transport.

### Keywords

Primary keywords were defined as symptoms or factors that describe the septic patient’s clinical presentation in the ED setting, e.g., “vomiting,” “fever,” or “hypotension.” The combined keywords consist of several primary and/or combined keywords. The primary and combined keywords were those identified in a previous study among Swedish EMS patients [5] with the addition of any new keyword describing the patient’s clinical presentation in the ED, i.e., not previously identified in the prior prehospital study [3].

### Study protocol

Keywords reflecting clinical presentation were quantified, both as primary keywords and as combined keywords within two subgroups of patients based on mode of arrival: by EMS or by means of transportation other than EMS.

ED and in-hospital records were reviewed (TakeCare® v. 18.3.10 CompuGroup Medical Stockholm Sweden) and analyzed with respect to prevalence of keywords mode of arrival and demographics (age gender and sepsis severity)

The chief complaint, current history, and preliminary assessment sections of the ED charts were analyzed for the quantification of keywords. The current history section focuses on the acute situation and not a comprehensive medical history. Only symptoms with new onset, defined as onset within 3 weeks prior to ED arrival, were considered relevant for the current study. Each patient could fulfill multiple primary and several different combined keywords.

### Data analysis

The statistical analysis program SPSS® (Statistical Package for Social Sciences SPSS®, IBM®, student version 22.0) was used to analyze the prevalence of keywords. Independent-samples *T* test was used to compare means and Chi-square test was used to compare proportions between patients arriving by EMS with those of patients arriving by other means (1) in the entire group of septic ED patients and (2) in strata based on age, gender, and severity of sepsis (severe vs non-severe sepsis) to adjust for possible differences between the EMS vs non-EMS groups due to these factors. Fischer’s exact test was used when the expected cell count was < 5.

*P* values are presented without adjustment for multiple comparisons and the Bonferroni-adjusted significance level is described in the footnote of each table by dividing the significance level 0.05 with the number of performed comparisons. Only two tailed *P* values that remained significant according to the Bonferroni-adjusted significance level are considered significant in the current study.

### Ethical approval

Stockholm Regional Ethical Review Board approval was obtained for this study (reference number 2012/1288-31/3).

## Results

### Patient characteristics

**Fig. 1:**
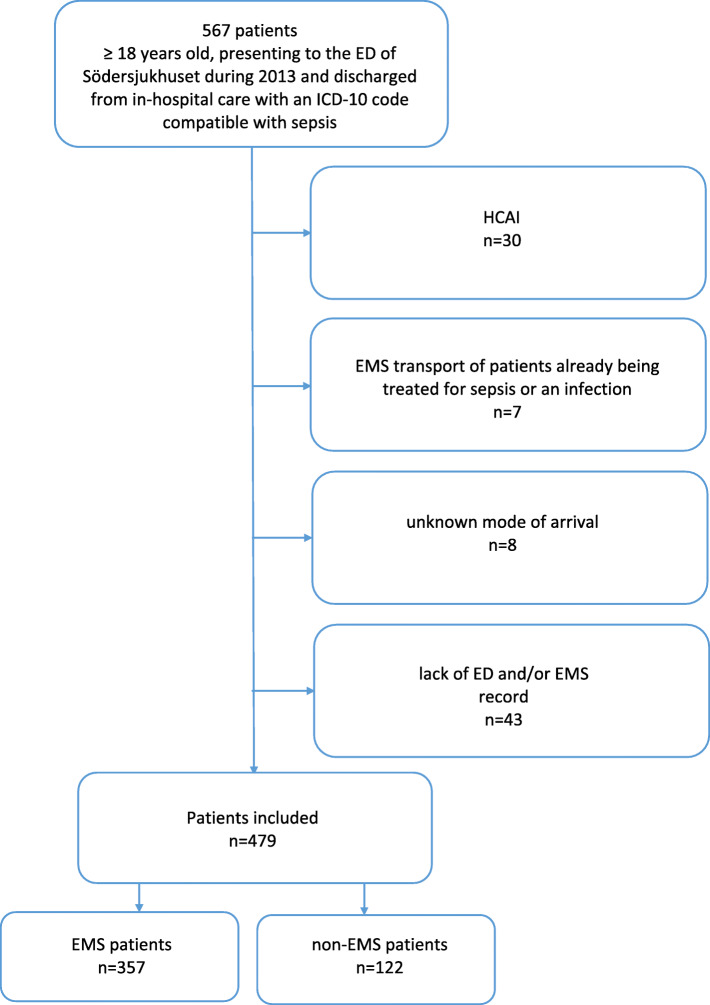
Flow chart for inclusion and exclusion of adult patients arriving to the ED of Södersjukhuset during 2013 and discharged with ICD code sepsis. EMS, emergency medical services; ED, emergency department; ICD, International Classification of Disease; HCAI, healthcare-associated infections

See Fig. 1 for flow chart for inclusion and exclusion of patients.

A total of 479 patients were included in current study: 357 EMS patients and 122 non-EMS patients.

Characteristics of the patients are presented in Table 1. The median age in the entire sample of septic ED patients was 75 years (Interquartile range, IQR 61-85), 255/479 (53.2%) were men and 248/459 (54.0%) with sufficient documentation to determine severity grade had severe sepsis. The overall in-hospital mortality was 99/479 (20.7%).

**Table 1:** Characteristics of the patients with community-acquired sepsis presenting to the ED of Södersjukhuset during 2013^*^

	Entire sample of septic patients, ***N*** = 479		EMS group, ***n*** = 357		non-EMS group, ***n*** = 122		***P*** value**
Variable	Median (IQR)	Number (%)	Median (IQR)	Number (%)	Median (IQR)	Number (%)	
**Age, years**	75 (61-85)		78.0 (68-86)		62.5 (39-73)		**< 0.001**
**Age groups**							
**< 65 years**		137/479 (28.6)		69/357 (19.3)		68/122 (55.7)	
**65-74 years**		102/479 (21.3)		76/357 (21.3)		26/122 (21.3)	
**≥ 75 years**		240/479 (50.1)		212/357 (59.4)		28/122 (23.0)	
**Gender**							
**Male**		255/479 (53.2)		196/357 (54.9)		59/122 (48.4)	0.211
**Severe sepsis**		248/459*** (54.0)		218/346*** (63.0)		30/113** (26.5)	**< 0.001**
**In-hospital mortality**		99/479 (20.7)		94/357 (26.3)		5/122 (4.1)	**< 0.001**

*ED* emergency department, *IQR* interquartile range, *EMS* emergency medical services

*The table illustrates characteristics of the entire population of patients admitted to the ED of Södersjukhuset during 2013 and discharged with an ICD-10 code compatible with sepsis, in addition to characteristics based on mode of arrival

***Number of patients with sufficient documentation to determine whether severe sepsis or not

The median age in the EMS group was 15.5 years older than in the non-EMS group (78.0 years; IQR 68-86 vs 62.5 years; IQR 39-73, *p* value < 0.001). Patients in the EMS group had a significantly higher prevalence of severe sepsis than patients in the non-EMS group (218/346; 63.0% vs 30/113; 26.5%, *p* value < 0.001) and a higher in-hospital mortality rate (94/357; 26.3% vs 5/122; 4.1%, *p* value < 0.001). There was no significant difference with respect to gender between the patients arriving by EMS vs non-EMS, see Table 1.

### Keywords

Ninety primary keywords and 14 combined keywords describing the presentation of septic patients were extracted from ED charts, see Additional files 2 and 3.

#### Primary keywords

The prevalence of primary keywords in the entire sample of septic patients and among patients arriving by EMS and non-EMS respectively is presented in Additional file 2.

Five new keywords were identified in the ED setting which were not present in the former study based in the prehospital setting [5]. These were “rash,” “sensitivity to sound,” “photosensitivity,” “blisters (on skin),” and “pale stool.” All five had a prevalence below or equal to 3/479 patients (0.6%), see Additional file 2.

#### Combined keywords

The prevalence of combined keywords in the entire sample of septic patients, and based on mode of arrival, is presented in Additional file 3.

#### All keywords exceeding a prevalence of 20%

The prevalence of all keywords (both primary and combined) exceeding 20% in the entire sample of septic patients and based on mode of arrival is presented in Table 2.

**Table 2:** Prevalence of keywords exceeding 20% among ED patients discharged with ICD-10 code sepsis^*^

Order	Keyword [5]	Prevalence						***P*** value**
		Entire sample of ED patients (***N*** = 479)		EMS group (***n*** = 357)		non-EMS group (***n*** = 122)		
		Number	Percent (%) and 95% CI	Number	Percent (%) and 95% CI	Number	Percent (%) and 95% CI	
**1**	**Abnormal, or suspected abnormal temperature** In turn including primary keywords shivering OR hypothermia OR the following combined keywords: Confirmed or suspected fever, confirmed abnormal temperature (confirmed fever or hypothermia)	319	66.6 (62.3-70.7)	232	65.0 (60.0-69.8)	87	71.3 (62.7-78.6)	0.201
**2**	**Pain** Abdominal/extremity/back/undefined/urinary tract/joint/chest/general/headache/throat/wound/painful muscle cramp/positive Pasternatsy’s sign (costovertebral angle tenderness)	230	48.0 (43.6-52.5)	143	40.1 (35.1-45.2)	87	71.3 (62.7-78.6)	**< 0.001**
**3**	**Abnormal breathing** Tachypnea, low oxygen saturation, airway secretions, breathing difficulties, cough, or obstructive breathing	210	43.8 (39.5-48.3)	185	51.8 (46.7-57.0)	25	20.5 (14.3-28.5)	**< 0.001**
**4**	**Risk factors for sepsis** Known ongoing or recent infection, current antibiotic treatment, recent invasive procedures, substance abuse, compromised immune system, chronically compromised breathing	172	35.9 (31.7-40.3)	110	30.8 (26.3-35.8)	62	50.8 (42.1-59.5)	**< 0.001**
**5**	**Abnormal circulation** Weak pulse or difficulties to palpate the pulse, peripheral coldness, cardiac arrest, tachycardia, low blood pressure, prolonged capillary refill time or non-measurable circulatory variables	163	34.0 (29.9-38.4)	137	38.4 (33.5-43.5)	26	21.3 (15.0-29.4)	**0.001**
**6**	**Temporal deterioration** Stated deterioration or expressions describing a temporal change	144	30.1 (26.1--34.3)	102	28.6 (24.1-33.5)	42	34.4 (26.6-43.2)	0.223
**7**	**Gastrointestinal symptoms** Vomiting, diarrhea, reduced amount of stool, gastrointestinal bleeding, obstipation, pale feces	137	28.6 (24.7-32.8)	104	29.1 (24.7-34.1)	33	27.0 (20.0-35.5)	0.660
**8**	**Acute altered mental status** Abnormal behavior or level of consciousness (excluding previously known dementia or mental retardation without statement worse) OR abnormal verbal response defined as no/decreased verbal response	127	26.5 (22.8-30.6)	111	31.1 (26.5-36.1)	16	13.1 (8.2-20.2)	**< 0.001**
**9**	**Abnormal skin** Paleness, wounds or wound infection, sweaty, cyanosis, redness, icterus, mottling, bruises, rash, blisters, or peteckiae, change of skin turgor, exuding skin	125	26.1 (22.4-30.2)	96	26.9 (22.6-31.7)	29	23.8 (17.1-32.1)	0.498
**10**	**Abnormal urination** Abnormal urination (such as hematuria without trauma, bad smelling or cloudy urine, increased frequency of urination) OR urinary tract pain OR decreased urinary volumes OR dysfunction of urinary catheters defined as obstruction/leakage/problematic urinary catheters including nefrostomias	118	24.6 (21.0-28.7)	92	25.8 (21.5-30.6)	26	21.3 (15.0-29.4)	0.324
**11**	**Loss of energy** Defined as fatigue, weakness, faintness or similar expressions	113	23.6 (20.0-27.6)	91	25.5 (21.3-30.3)	22	18.0 (12.2-25.8)	0.094
**12**	**Decreased mobility** in turn including primary keywords remained sitting or lying in an abnormal way OR decreased miscellaneous mobility OR the following combined keywords: “weakness of the legs” and “fallen or being found on the floor”	106	22.1 (18.6-26.1)	93	26.1 (21.8-30.8)	13	10.7 (6.3-17.4)	**< 0.001**

*ED* emergency department, *EMS* emergency medical services, *CI* confidence interval

*The prevalence of all keywords (both primary and combined) exceeding 20% in the entire sample of patients admitted to the ED of Södersjukhuset during 2013 and discharged with an ICD-10 code compatible with sepsis. The table illustrates the prevalence in the entire group and the prevalence based on mode of arrival

**For comparison between EMS and non-EMS groups. *P* values are presented without adjustment for multiple comparisons. In total 13 tests were performed. Bonferroni-adjusted significance level is 0,05/13 = 0,0038. *P* values indicating significant differences after adjustment for multiple comparisons by Bonferroni correction are bolded and considered significant in the current study

##### All septic patients

Twelve keywords (primary and combined) reflecting septic patients’ presentation exceeded a prevalence of 20% in the entire sample of 479 patients: “abnormal, or suspected abnormal temperature” (*n* = 319, 66.6%), “pain” (*n* = 230, 48.0%), “abnormal breathing” (*n* = 210, 43.8%), “risk factors for sepsis” (*n* = 172, 35.9%), “abnormal circulation” (*n* = 163, 34.0%), “temporal deterioration” (*n* = 144, 30.1%), “gastrointestinal symptoms” (*n* = 137, 28.6%), “acute altered mental status” (*n* = 127, 26.5%), “abnormal skin” (*n* = 125, 26.1%), “abnormal urination” (*n* = 118 , 24.6%), “loss of energy” (*n* = 113, 23.6%), and “decreased mobility” (*n* = 106, 22.1%), see Table 2.

##### Comparison between all keywords with a prevalence exceeding 20%

Four keywords (primary and combined) with a prevalence exceeding 20% in the entire sample of septic patients were significantly more frequent in the EMS group than in the non-EMS group: “abnormal breathing” (185/357; 51.8% vs 25/122; 20.5%, *p* value < 0.001),“abnormal circulation” (137/357; 38.4% vs 26/122; 21.3%, *p* value < 0.001), “acute altered mental status” (111/357; 31.1% vs 16/122; 13.1%, *p* value < 0.001) and “decreased mobility” (93/357; 26.1% vs 13/122; 10.7%, *p* value < 0.001), see Table 2.

Two keywords with a prevalence exceeding 20% in the entire sample of septic patients were significantly more frequent in the non-EMS group than in the EMS group: “pain” (87/122; 71.3% vs 143/357; 40.1%, *p* value < 0.001) and “risk factors for sepsis” (62/122; 50.8% vs 110/357; 30.8%, *p* value < 0.001), see Table 2.

Stratification analyses based on age, gender, and sepsis severity are presented in Additional files 4, 5, and 6.

## Discussion

The most frequent keywords reflecting clinical presentation in the entire sample of septic patients arriving to the ED were “abnormal, or suspected abnormal temperature,” “pain,” “abnormal breathing,” “risk factors for sepsis,” “abnormal circulation,” “temporal deterioration,” “gastrointestinal symptoms,” “acute altered mental status,” “abnormal urination,” “loss of energy,” and “decreased mobility.” Keywords more common among septic patients arriving by EMS were “abnormal breathing,” “abnormal circulation,” “acute altered mental status,” and “decreased mobility” while “pain” and “risk factors for sepsis” were more frequent among septic patients arriving to the ED by means other than EMS.

### Keywords with a similar prevalence among patients arriving by EMS and non-EMS

The most common combined keyword among both patients arriving by EMS and by non-EMS was “abnormal, or suspected abnormal temperature.” Both fever and hypothermia have previously been described as symptoms related to the septic patient [3, 13]. However, despite “abnormal, or suspected abnormal temperature” being the most prevalent keyword in the current study, approximately one-third of the included patients did not present with fever. This finding is consistent with previous studies [3, 14].

“Temporal deterioration” represents an acute change in the patient’s habitual state, but it does not describe the details of this change. The prevalence of temporal deterioration was high both among EMS and non-EMS patients. These findings are consistent with those demonstrated by Bohm et al. in emergency calls involving septic patients [15].

Vomiting and diarrhea were common among both patients arriving by EMS and by non-EMS. The high frequency of “gastrointestinal symptoms” in the current study is supported by previous studies [3, 13].

The keywords “abnormal skin,” “abnormal urination,” and “loss of energy” were also present among patients arriving by both EMS and by non-EMS to a similar extent.

### Keywords more frequent among patients arriving by EMS

“Abnormal circulation,” “abnormal breathing,” “acute altered mental status,” and “decreased mobility” were all significantly more common among patients arriving by EMS. The first three are directly connected to the former criteria for severe sepsis [11] and the findings most likely reflect that EMS patients are more severely ill than non-EMS patients, which is also supported by previous studies [8].

### Keywords more frequent among patients not arriving by EMS

“Pain” is an unspecific symptom and the single most common chief complaint among ED patients in general [16]. That pain was more common among non-EMS patients could potentially be a consequence of the increased prevalence of acute altered mental status among patients arriving by EMS, in turn impairing the patient’s capability to express pain. Furthermore, keywords reflecting abnormal vital signs were more common among patients arriving by EMS patients and abnormal vital signs may render more attention among emergency care providers as compared to symptoms, e.g., pain.

The combined keyword “risk factors for sepsis” includes several primary keywords such as ongoing or recent infection/invasive procedures/immunosuppressive treatment but did not include traditional risk factors for sepsis such as age and pre-existing comorbidity. However, traditional risk factors should be considered in a future screening tool. The current study included components registered in the chief complaint, current history, and preliminary assessment sections of the ED charts, where the current history section focuses on the acute situation and not a comprehensive medical history, e.g., pre-existing comorbidity. Patients with “risk factors for sepsis,” as defined in the current study, are often informed to seek medical attention if they deteriorate and may therefore seek medical attention at an earlier stage and hence need an ambulance to a lesser extent. In addition, patients with “risk factors for sepsis” have previously been shown to be younger [3] which may affect the capacity to use means of transportation other than the ambulance, but these speculations remain to be investigated.

### Reflections on observed differences between patients arriving by EMS vs by non-EMS and clinical implementation of the results

Several factors are thought to contribute to the observed differences in keyword prevalence based on mode of arrival. We do not believe that the arrival mode per se exhibits causality on the presentation but rather that the observed differences reflect that patients arriving by EMS are older and have a higher prevalence of severe sepsis as compared with patients arriving by other means. This is supported by the stratification analyses showing that “abnormal circulation,” “acute altered mental status,” and “decreased mobility” did not remain significantly more frequent among EMS patients and “risk factors for sepsis” did not remain significantly more frequent among non-EMS patients when stratified for sepsis severity, indicating that differences in sepsis severity accounts for the observed differences for these keywords between EMS vs non-EMS patients.

The observed differences between septic EMS vs non-EMS patients support the principle of adapting sepsis screening tools to the population where they are planned to be implemented; in this case, the EMS vs the ED, and can be exemplified as a screening tool applied in the EMS may include variables, e.g., “abnormal circulation,” “abnormal breathing,” “acute altered mental status,” and “decreased mobility,” while a screening tool proposed to be used for “walk-in” ED patients may include other variables such as “pain” and “risk factors for sepsis.”

Nevertheless, despite these observed differences, we would like to emphasize that most keywords demonstrated a similar distribution regardless of mode of arrival and the most prevalent keywords related to sepsis presentation in the current study confirm prior results from the prehospital [3, 15] and ED settings [13].

There are several limitations to the current study.

The definition of sepsis based on ICD-code can be questioned, as it is well known that identification of septic patients based on ICD codes leads to an underestimation of the true number of septic patients [17] and hence, there is a risk that the study sample may not be representative of all ED patients with sepsis. Inclusion based on ICD-code sepsis may involve a selection of the most severely ill septic patients. However, the method has been used in large epidemiological studies of sepsis [18] and is the only realistic method for larger registry studies.

Furthermore, the current study was a retrospective study with the inherent limitations of missing data. The prevalence of keywords was based on documented observations in ED records. Documentation may depend on various factors such as inter-individual variation among ED personnel and ED workload. Many of the keywords represent symptoms, and the identification of symptoms requires a thorough history taking by the ED doctor in addition to a communicable patient.

Multiple comparisons were performed, with the inherent risk of inferring type I errors. Therefore, the level of significance was adjusted by applying a Bonferroni correction. However, the Bonferroni correction is excessively strict with the inherent risk of inferring type II errors [19]. Hence, although erring on the side of caution, this may have resulted in true differences being regarded as non-significant.

Furthermore, when comparing the prevalence of especially primary keywords, the number of patients in the compared groups was few and hence the results must be interpreted with caution and need to be confirmed in larger samples.

Finally, the current study is a single center study which may limit the generalizability of the results. However, patients were included over a period of 1 year which enables the seasonal variation of sepsis to be accounted for. In addition, the study setting was the largest ED in Scandinavia at the time [9].

## Conclusions

The distribution of most keywords related to sepsis presentation was similar irrespective of mode of arrival, however, some differences were present. Keywords “abnormal breathing,” “abnormal circulation,” “acute altered mental status,” and “decreased mobility” were more common among patients arriving by EMS while “pain” and “risk factors for sepsis” were more common among patients arriving by means other than EMS. The results indicate that septic patients arriving to the ED via EMS are older and more often have severe sepsis. This information may be useful in clinical decision tools or sepsis screening tools but needs to be evaluated in prospective studies.

## Supplementary Information

Additional file 1: (PDF). Definition of severe sepsis.

Additional file 2: (PDF). Prevalence of primary keywords. The prevalence of primary keywords in the entire sample of septic patients presenting to Södersjukhuset’s emergency department during 2013 and prevalence based on mode of arrival.

Additional file 3: (PDF). Prevalence of combined keywords. The prevalence of combined keywords in the entire sample of septic patients presenting to Södersjukhuset´s emergency department during 2013 and prevalence based on mode of arrival.

Additional file 4: (PDF). Prevalence of keywords exceeding 20% based on mode of arrival and age group. A comparison of prevalence of all keywords* exceeding 20% among septic patients presenting to Södersjukhuset´s emergency department during 2013, based on mode of arrival AND age group.

Additional file 5: (PDF). Prevalence of keywords exceeding 20% based on mode of arrival and gender. A comparison of prevalence of all keywords* exceeding 20% among septic patients presenting to Södersjukhuset´s emergency department during 2013, based on mode of arrival AND gender.

Additional file 6: (PDF). Prevalence of keywords exceeding 20% based on mode of arrival and sepsis severity. A comparison of prevalence of all keywords* exceeding 20% among septic patients presenting to Södersjukhuset´s emergency department during 2013, based on mode of arrival AND sepsis severity.

## Data Availability

The data that support the findings of this study are available from Örebro University, but restrictions apply to the availability of these data, which were used under license for the current study, and so are not publicly available. Data are however available from the authors upon reasonable request and with permission from Örebro University.

## Abbreviations

EMS: Emergency medical services
ED: Emergency department
ICD: International Classification of Diseases
HCAI: Healthcare-associated infection
SPSS: Statistical Package for the Social Sciences
IQR: Interquartile range
