# Supplementary material for: Keywords reflecting sepsis presentation based on mode of emergency department arrival: a retrospective cross-sectional study
Source: Int J Emerg Med. 2021 Dec 20;14:78. doi: 10.1186/s12245-021-00396-z (PMC8903703; doi:10.1186/s12245-021-00396-z)
Supplement: Supplementary file 1 — (PDF). Definition of severe sepsis. [file 12245_2021_396_MOESM1_ESM.pdf]

Additional file 1. Definition of severe sepsis

Severe sepsis was defined as fulfillment of one or more of the following criteria in the ED; a systolic blood pressure <90 mmHg [1], an oxygen saturation of ≤86% if the lung was not focus of infection or an oxygen saturation ≤78% if the lung was focus of infection [2], an acute altered mental status [2], mottling [1] or cardiopulmonary arrest due to sepsis (but admitted alive to in-hospital care).

References:

1. Levy MM, Fink MP, Marshall JC, Abraham E, Angus D, Cook D, et al. 2001 SCCM/ESICM/ACCP/ATS/SIS International Sepsis Definitions Conference. Crit Care Med. 2003;31(4):1250-6.
2. Infektionsläkarföreningen S**.** Vårdprogram Svår sepsis och septisk chock -tidig identifiering och initial handläggning. 2012.
