# Supplementary material for: Keywords reflecting sepsis presentation based on mode of emergency department arrival: a retrospective cross-sectional study
Source: Int J Emerg Med. 2021 Dec 20;14:78. doi: 10.1186/s12245-021-00396-z (PMC8903703; doi:10.1186/s12245-021-00396-z)
Supplement: Supplementary file 2 — (PDF). Prevalence of primary keywords. The prevalence of primary keywords in the entire sample of septic patients presenting to Södersjukhuset’s emergency department during 2013 and prevalence based on mode of arrival. [file 12245_2021_396_MOESM2_ESM.pdf]

Additional file 2. Prevalence of primary keywords.

The prevalence of primary keywords [1] in the entire sample of septic patients presenting to the emergency department of Södersjukhuset emergency during 2013 and prevalence based on mode of arrival.

		**Prevalence**						
		**Entire sample (N=479)**		**EMS patients (n=357)**		**non-EMS patients (n=122)**		
**Order**	**Primary keyword [1]**	**Number**	**Percent (%) and 95% CI**	**Number**	**Percent (%) and 95% CI**	**Number**	**Percent (%) and 95% CI**	**P-value***
**1**	**Confirmed fever** Statement fever or statement temperature >38˚	271	56.6 (52.1-61.0)	198	55.5 (50.3-60.5)	73	59.8 (51.0-68.1)	0.400
**2**	**Temporal deterioration** Stated deterioration or expressions describing a temporal change	144	30.1 (26.1-34.3)	102	28.6 (24.1-33.5)	42	34.4 (26.6-43.2)	0.223
**3**	**Loss of energy** Defined as fatigue, weakness, faintness or similar expressions	113	23.6 (20.0-27.6)	91	25.5 (21.3-30.3)	22	18.0 (12.2-25.8)	0.094
**4**	**Low blood pressure** Statement systolic blood pressure ≤90 mmHg	113	23.6 (20.0-27-6)	97	27.2 (22.8-32.0)	16	13.1 (8.2-20.2)	0.002
**5**	**Breathing difficulties** Statement difficulties to breath, dyspnea, shortness of breath, shallow breathing or similar expressions	107	22.3 (18.8-26.3)	97	27.2 (22.8-32.0)	10	8.2 (4.5-14.4)	**0.000**
**6**	**Shivering**	96	20.0 (16.7-23.9)	59	16.5 (13.0-20.7)	37	30.3 (22.9-39.0)	0.001
**7**	**Decreased general condition** Including expressions such as poor general condition, affected general condition	94	19.6 (16.3-23.4)	84	23.5 (19.4-28.2)	10	8.2 (4.5-14.4)	**0.000**
**8**	**Recent invasive procedures** Including IV drug abuse, surgical and urological procedures, new IV or urinary catheters	93	19.4 (16.1-23.2)	50	14.0 (10.8-18.0)	43	35.2 (27.3-44.1)	**0.000**
**9**	**Abdominal pain**	92	19.2 (15.9-23.0)	59	16.5 (13.0-20.7)	33	27.0 (20.0-35.5)	0.011

**10**	**Abnormal behaviour or level of consciousness** excluding abnormal verbal response	87	18.2 (15.0-21.9)	74	20.7 (16.9-25.2)	13	10.7 (6.3-17.4)	0.004
**11**	**Tachypnea** Statement tachypnea, rapid breathing, high respiratory rate, respiratory rate >20 or similar expressions	84	17.5 (14.4-21.2)	79	22.1 (18.1-26.7)	5	4.1 (1.8-9.2)	**0.000**
**12**	**Abnormal verbal response** Defined as no/decreased/changed verbal response	80	16.7 (13.6-20.3)	71	19.9 (16.1-24.3)	9	7.4 (3.9-13.4)	**0.000**
**13**	**Abnormal micturition** Defined as haematuria without trauma, bad smelling or cloudy urine, increased frequency of urination	76	15.9 (12.9-19.4)	56	15.7 (12.3-19.8)	20	16.4 (10.9-24.0)	0.854
**14**	**Tachycardia** Statement heart rate >90/min or expressions such as rapid heart rate, rapid pulse or similar expressions	74	15.5 (12.5-19.0)	66	18.5 (14.8-22.9)	8	6.6 (3.4-12.4)	0.002
**15**	**Reduced intake of food, fluids or oral medicines** Including reduced/no appetite	73	15.2 (12.3-18.7)	55	15.4 (12.0-19.5)	18	14.8 (9.5-22.1)	0.863
**16**	**Vomiting**	71	14.8 (11.9-18.3)	54	15.1 (11.8-19.2)	17	13.9 (8.9-21.2)	0.749
**17**	**Known ongoing or recent infection**	66	13.8 (11.0-17.2)	50	14.0 (10.8-18.0)	16	13.1 (8.2-20.2)	0.805
**18**	**Low oxygen saturation** Defined as statement oxygen saturation <90%	65	13.6 (10.8-16.9)	61	17.1 (13.5-21.3)	4	3.3 (1.3-8.1)	**0.000**
**19**	**Cough**	57	11.9 (9.3-15.1)	41	11.5 (8.6-15.2)	16	13.1 (8.2-20.2)	0.631
**20**	**Wounds or wound infection**	55	11.5 (8.9-14.7)	42	11.8 (8.8-15.5)	13	10.7 (6.3-17.4)	0.740
**21**	**Diarrhoea**	52	10.9 (8.4-14.0)	41	11.5 (8.6-15.2)	11	9.0 (5.1-15.4)	0.449
**22**	**Current antibiotic treatment**	50	10.4 (8.0-13.5)	39	10.9 (8.1-14.6)	11	9.0 (5.1-15.4)	0.552
**23**	**Airway secretions** Including expectorations, crackles and similar expressions	48	10.0 (7.6-13.0)	43	12.0 (9.1-15.8)	5	4.1 (1.8-9.2)	0.012

**24**	**Nausea**	46	9.6 (7.3-12.6)	26	7.3 (5.0-10.5)	20	16.4 (10.9-24.0)	0.003
**25**	**Decreased ability to stand or walk**<br>Including need to carry/lift the patient	46	9.6 (7.3-12.6)	35	9.8 (7.1-13.3)	11	9.0 (5.1-15.4)	0.799
**26**	**Oedema/Swelling**	44	9.2 (6.9-12.1)	31	8.7 (6.2-12.1)	13	10.7 (6.3-17.4)	0.515
**27**	**Fallen**	44	9.2 (6.9-12.1)	42	11.8 (8.8-15.5)	2	1.6 (0.5-5.8)	0.001
**28**	**Substance abuse**<br>Defined as drug abuse, alcohol overconsumption and all other terms indicating substance abuse such as "lives in a home for addicts", "patient at an outdoor clinic for substance abuse"	43	9.0 (6.7-11.9)	35	9.8 (7.1-13.3)	8	6.6 (3.4-12.4)	0.279
**29**	**Reduced urinary volumes**	38	7.9 (5.8-10.7)	37	10.4 (7.6-14.0)	1	0.8 (0.1-5.0)	0.001
**30**	**Extremity pain**	35	7.3 (5.3-10.0)	21	5.9 (3.9-8.8)	14	11.5 (7.0-18.4)	0.040
**31**	**Back pain**	32	6.7 (4.8-9.3)	22	6.2 (4.1-9.2)	10	8.2 (4.5-14.4)	0.437
**32**	**Suspected fever**<br>Defined as statement feeling hot/warm, increasing temperature or similar expressions	29	6.1 (4.2-8.6)	18	5.0 (3.2-7.8)	11	9.0 (5.1-15.4)	0.112
**33**	**Malaise**<br>Defined as expressions such as feeling sick, feeling bad, not feeling well and similar expressions	27	5.6 (3.9-8.1)	14	3.9 (2.4-6.5)	13	10.7 (6.3-17.4)	0.005
**34**	**Urinary tract pain**	27	5.6 (3.9-8.1)	15	4.2 (2.6-6.8)	12	9.8 (5.7-16.4)	0.020
**35**	**Compromised immune system**<br>Chemotherapy or other immunosuppressive treatment	26	5.4 (3.7-7.8)	19	5.3 (3.4-8.2)	7	5.7 (2.8-11.4)	0.861
**36**	**Joint pain**	25	5.2 (3.6-7.6)	11	3.1 (1.7-5.4)	14	11.5 (7.0-18.4)	**0.000**
**37**	**Undefined pain**	25	5.2 (3.6-7.6)	16	4.5 (2.8-7.2)	9	7.4 (3.9-13.4)	0.215
**38**	**Chest pain**	24	5.0 (3.4-7.4)	13	3.6 (2.1-6.1)	11	9.0 (5.1-15.4)	0.019
**39**	**Focal neurological findings**	24	5.0 (3.4-7.4)	21	5.9 (3.9-8.8)	3	2.5 (0.8-7.0)	0.135
**40**	**Found on the floor**<br>or corresponding place	24	5.0 (3.4-7.4)	24	6.7 (4.6-9.8)	0	0.0 (0.0-3.1)	0.003
**41**	**General pain**	23	4.8 (3.2-7.1)	13	3.6 (2.1-6.1)	10	8.2 (4.5-14.4)	0.042

**42**	**Redness (of skin)**	22	4.6 (3.1-6.9)	11	3.1 (1.7-5.4)	11	9.0 (5.1-15.4)	0.007
**43**	**High CRP**<br>Taken previous to ED arrival	22	4.6 (3.1-6.9)	13	3.6 (2.1-6.1)	9	7.4 (3.9-13.4)	0.089
**44**	**Reduced amount of stool**	21	4.4 (2.9-6.6)	21	5.9 (3.9-8.8)	0	0.0 (0.0-3.1)	0.006
**45**	**Positive Pasternatsky´s sign**<br>Costovertebral angle tenderness	19	4.0 (2.6-6.1)	9	2.5 (1.3-4.7)	10	8.2 (4.5-14.4)	0.012 (F*)
**46**	**Headache**	18	3.8 (2.4-5.9)	7	2.0 (1.0-4.0)	11	9.0 (5.1-15.4)	0.001 (F*)
**47**	**Remained lying or sitting**<br>Statement of being remained sitting or lying in an abnormal way	18	3.8 (2.4-5.9)	15	4.2 (2.6-6.8)	3	2.5 (0.8-7.0)	0.582 (F*)
**48**	**Gastrointestinal bleeding**<br>Including melena, hematemesis, hematochezia	18	3.8 (2.4-5.9)	16	4.5 (2.8-7.2)	2	1.6 (0.5-5.8)	0.267 (F*)
**49**	**Non-measurable circulatory variables**	18	3.8 (2.4-5.9)	17	4.8 (3.0-7.5)	1	0.8 (0.1-5.0)	0.053 (F*)
**50**	**Hypothermia**<br>Defined as statement hypothermia or "very low temp" or statement temperature <36°	16	3.3 (2.1-5.4)	14	3.9 (2.4-6.5)	2	1.6 (0.5-5.8)	0.380 (F*)
**51**	**Irregular pulse**	16	3.3 (2.1-5.4)	16	4.5 (2.8-7.2)	0	0.0 (0.0-3.1)	0.016 (F*)
**52**	**High blood sugar**<br>Plasma Glucose >12 mmol/L, regardless diabetes or not.	14	2.9 (1.8-4.8)	14	3.9 (2.4-6.5)	0	0.0 (0.0-3.1)	0.026 (F*)
**53**	**Anxiety or fear**	12	2.5 (1.4-4.3)	8	2.2 (1.1-4.4)	4	3.3 (1.3-8.1)	0.512 (F*)
**54**	**Fainting-but now awake**	12	2.5 (1.4-4.3)	10	2.8 (1.5-5.1)	2	1.6 (0.5-5.8)	0.739 (F*)
**55**	**Sweaty**	11	2.3 (1.3-4.1)	9	2.5 (1.3-4.7)	2	1.6 (0.5-5.8)	0.737 (F*)
**56**	**Dysarthria**<br>Slurred speech (but non-affected level of consciousness)	11	2.3 (1.3-4.1)	9	2.5 (1.3-4.7)	2	1.6 (0.5-5.8)	0.737 (F*)
**57**	**Dysfunction of urinary catheters**<br>Including obstruction/leakage/problematic urinary catheters including nefrostomias	10	2.1 (1.1-3.8)	8	2.2 (1.1-4.4)	2	1.6 (0.5-5.8)	1.000 (F*)
**58**	**Weak pulse or difficulties to palpate the pulse**	10	2.1 (1.1-3.8)	10	2.8 (1.5-5.1)	0	0.0 (0.0-3.1)	0.072 (F*)

**59**	**Soiled patient** Patient wetted from his/her own urine or stool	10	2.1 (1.1-3.8)	10	2.8 (1.5-5.1)	0	0.0 (0.0-3.1)	0.072 (F*)
**60**	**Obstipation**	9	1.9 (1.0-3.5)	0	0.0 (0.0-1.1)	9	7.4 (3.9-13.4)	**0.000 (F*)**
**61**	**Dizziness**	9	1.9 (1.0-3.5)	6	1.7 (0.8-3.6)	3	2.5 (0.8-7.0)	0.699 (F*)
**62**	**Bloodstained patient**	8	1.7 (0.9-3.3)	6	1.7 (0.8-3.6)	2	1.6 (0.5-5.8)	1.000 (F*)
**63**	**Peripheral coldness**	8	1.7 (0.9-3.3)	8	2.2 (1.1-4.4)	0	0.0 (0.0-3.1)	0.212 (F*)
**64**	**Seizures**	8	1.7 (0.9-3.3)	8	2.2 (1.1-4.4)	0	0.0 (0.0-3.1)	0.212 (F*)
**65**	**High blood pressure** Statement high blood pressure or statement blood pressure ≥160 mmHg systolic or ≥100 mmHg diastolic	7	1.5 (0.7-3.0)	4	1.1 (0.4-2.8)	3	2.5 (0.8-7.0)	0.378 (F*)
**66**	**Paleness**	7	1.5 (0.7-3.0)	7	2.0 (1.0-4.0)	0	0.0 (0.0-3.1)	0.199 (F*)
**67**	**History of positive findings in blood culture** Positive blood culture taken previous to EMS arrival-during a visit to the hospital or by other health care provider but the patient is now at home	6.	1.3 (0.6-2.7)	1	0.3 (0.1-1.6)	5	4.1 (1.8-9.2)	0.005 (F*)
**68**	**Throat pain**	5	1.0 (0.4-2.4)	3	0.8 (0.3-2.4)	2	1.6 (0.5-5.8)	0.605 (F*)
**69**	**Mottling**	5	1.0 (0.4-2.4)	5	1.4 (0.6-3.2)	0	0.0 (0.0-3.1)	0.336 (F*)
**70**	**Cyanosis** Including blue fingers/nails/lips/toes	4	0.8 (0.3-2.1)	4	1.1 (0.4-2.8)	0	0.0 (0.0-3.1)	0.576 (F*)
**71**	**Obstructive breathing**	4	0.8 (0.3-2.1)	4	1.1 (0.4-2.8)	0	0.0 (0.0-3.1)	0.576 (F*)
**72**	**Bruises or peteckiae**	4	0.8 (0.3-2.1)	4	1.1 (0.4-2.8)	0	0.0 (0.0-3.1)	0.576 (F*)
**73**	**Rash (on skin)**	3	0.6 (0.2-1.8)	0	0.0 (0.0-1.1)	3	2.5 (0.8-7.0)	0.016 (F*)
**74**	**Dry mucous membranes (of the mouth)**	3	0.6 (0.2-1.8)	3	0.8 (0.3-2.4)	0	0.0 (0.0-3.1)	0.575 (F*)
**75**	**Wound pain**	2	0.4 (0.1-1.5)	0	0.0 (0.0-1.1)	2	1.6 (0.5-5.8)	0.064 (F*)
**76**	**Photosensitivity**	2	0.4 (0.1-1.5)	0	0.0 (0.0-1.1)	2	1.6 (0.5-5.8)	0.064 (F*)
**77**	**Blisters (on skin)**	2	0.4 (0.1-1.5)	0	0.0 (0.0-1.1)	2	1.6 (0.5-5.8)	0.064 (F*)
**78**	**Pale stool**	2	0.4 (0.1-1.5)	0	0.0 (0.0-1.1)	2	1.6 (0.5-5.8)	0.064 (F*)

**79**	**Chronically compromised breathing** Such as painful conditions or neurological diseases compromising breathing	2	0.4 (0.1-1.5)	1	0.3 (0.1-1.6)	1	0.8 (0.1-5.0)	0.445 (F*)
**80**	**Non-measurable breathing variables**	2	0.4 (0.1-1.5)	2	0.6 (0.2-2.0)	0	0.0 (0.0-3.1)	1.000 (F*)
**81**	**Icterus**	2	0.4 (0.1-1.5)	2	0.6 (0.2-2.0)	0	0.0 (0.0-3.1)	1.000 (F*)
**82**	**Decreased miscellaneous mobility** Including expressions such as stiffness when trying to move arms, disability to sit or disability to squeeze the investigators hand	2	0.4 (0.1-1.5)	2	0.6 (0.2-2.0)	0	0.0 (0.0-3.1)	1.000 (F*)
**83**	**Cardiac arrest**	2	0.4 (0.1-1.5)	2	0.6 (0.2-2.0)	0	0.0 (0.0-3.1)	1.000 (F*)
**84**	**Sensitivity to sound**	1	0.2 (0.0-1.2)	0	0.0 (0.0-1.1)	1	0.8 (0.1-5.0)	0.255 (F*)
**85**	**Change of skin turgor**	1	0.2 (0.0-1.2)	1	0.3 (0.1-1.6)	0	0.0 (0.0-3.1)	1.000 (F*)
**86**	**Exuding skin**	1	0.2 (0.0-1.2)	1	0.3 (0.1-1.6)	0	0.0 (0.0-3.1)	1.000 (F*)
**87**	**Prolonged capillary refill time**	1	0.2 (0.0-1.2)	1	0.3 (0.1-1.6)	0	0.0 (0.0-3.1)	1.000 (F*)
**88**	**Painful muscle cramp**	1	0.2 (0.0-1.2)	1	0.3 (0.1-1.6)	0	0.0 (0.0-3.1)	1.000 (F*)
**89**	**Palpitations**	1	0.2 (0.0-1.2)	1	0.3 (0.1-1.6)	0	0.0 (0.0-3.1)	1.000 (F*)
**90**	**Feeling of depression**	0	0.0 (0.0-0.8)	0	0.0 (0.0-1.1)	0	0.0 (0.0-3.1)	-

EMS= Emergency Medical Services, CI=Confidence Interval, IV= Intravenous, CRP= C-Reactive Protein

*for comparison between EMS and non-EMS patients. P-values are presented without adjustment for multiple comparisons. In total 90 tests were performed. Bonferroni-adjusted significance level is 0,05/90=0,00056. P-values indicating significant differences after adjustment for multiple comparisons by Bonferroni correction are bolded and considered significant in the current study.

F* Fischer's exact test was used for statistical analysis due to expected count being <5.

References:
1. Wallgren UM, Bohm KEM, Kurland L. Presentations of adult septic patients in the prehospital setting as recorded by emergency medical services: a mixed methods analysis. Scand J Trauma Resusc Emerg Med. 2017;25(1):23.
