# Supplementary material for: Keywords reflecting sepsis presentation based on mode of emergency department arrival: a retrospective cross-sectional study
Source: Int J Emerg Med. 2021 Dec 20;14:78. doi: 10.1186/s12245-021-00396-z (PMC8903703; doi:10.1186/s12245-021-00396-z)
Supplement: Supplementary file 3 — (PDF). Prevalence of combined keywords. The prevalence of combined keywords in the entire sample of septic patients presenting to Södersjukhuset´s emergency department during 2013 and prevalence based on mode of arrival. [file 12245_2021_396_MOESM3_ESM.pdf]

Additional file 3. Prevalence of combined keywords.

The prevalence of combined keywords [1] in the entire population of septic patients presenting to the emergency department of Södersjukhuset during 2013 and prevalence based on mode of arrival.

		Prevalence						
		Entire sample (N=479)		EMS patients (n=357)		non-EMS patients (n=122)		
Order	Combined keyword [1]	Number	Percent (%) and 95% CI	Number	Percent (%) and 95% CI	Number	Percent (%) and 95% CI	P-value*
1	**Abnormal, or suspected abnormal temperature** In turn including primary keywords shivering OR hypothermia OR the following combined keywords:	319	66.6 (62.3-70.7)	232	65.0 (60.0-69.8)	87	71.3 (62.7-78.6)	0.201
	**-Confirmed or suspected fever** Fever defined as statement fever or statement temperature >38˚ OR suspected fever defined as statement feeling hot/warm, increasing temperature or similar expressions	296	61.8 (57.4-66.0)	214	59.9 (54.8-64.9)	82	67.2 (58.5-74.9)	0.154
	**-Confirmed abnormal temperature (confirmed fever or hypothermia)** Statement fever or statement temperature >38˚ OR statement hypothermia or temperature <36˚	286	60.0 (55.3-64.0)	212	59.4 (54.2-64.4)	74	60.7 (51.8-68.9)	0.805
2	**Pain** Abdominal/extremity/back/ undefined/urinary tract/joint/chest/general/ headache/throat/wound/painful muscle cramp/positive Pasternatsy´s sign (costovertebral angle tenderness)	230	48.0 (43.6-52.5)	143	40.1 (35.1-45.2)	87	71.3 (62.7-78.6)	**<0.001**
3	**Abnormal breathing** Tachypnea, low oxygen saturation, airway secretions, breathing difficulties, cough, or obstructive breathing	210	43.8 (39.5-48.3)	185	51.8 (46.7-57.0)	25	20.5 (14.3-28.5)	**<0.001**
4	**Risk factors for sepsis** Known ongoing or recent infection, current antibiotic treatment, recent invasive procedures, substance abuse, compromised immune system), chronically compromised breathing	172	36.0 (31.7-40.3)	110	30.8 (26.3-35.8)	62	50.8 (42.1-59.5)	**<0.001**

**5**	**Abnormal circulation** Weak pulse or difficulties to palpate the pulse, peripheral coldness, cardiac arrest, tachycardia, low blood pressure, prolonged capillary refill time or non-measurable circulatory variables	163	34.0 (29.9-38.4)	137	38.4 (33.5-43-5)	26	21.3 (15.0-29.4)	**0.001**
**6**	**Gastrointestinal symptoms** Vomiting, diarrhoea, reduced amount of stool, gastrointestinal bleeding, obstipation, pale faeces	137	28.6 (24.7-32.8)	104	29.1 (24.7-34.1)	33	27.0 (20.0-35.5)	0.660
**7**	**Acute altered mental status** Abnormal behaviour or level of consciousness (excluding previously known dementia or mental retardation without statement worse) OR abnormal verbal response defined as no/decreased verbal response	127	26.5 (22.8-30.6)	111	31.1 (26.5-36.1)	16	13.1 (8.2-20.2)	**<0.001**
**8**	**Abnormal skin** Paleness, wounds or wound infection, sweaty, cyanosis, redness, icterus, mottling, bruises, rash, blisters or peteckiae, change of skin turgor, exuding skin	125	26.1 (22.4-30.2)	96	26.9 (22.6-31.7)	29	23.8 (17.1-32.1)	0.498
**9**	**Abnormal urination** Abnormal urination (such as haematuria without trauma, bad smelling or cloudy urine, increased frequency of urination) OR urinary tract pain OR decreased urinary volumes OR dysfunction of urinary catheters defined as obstruction/leakage/problematic urinary catheters including nefrostomias	118	24.6 (21.0-28.7)	92	25.8 (21.5-30.6)	26	21.3 (15.0-29.4)	0.324
**10**	**Decreased mobility** In turn including primary keywords remained sitting or lying in an abnormal way OR decreased miscellaneous mobility OR the following combined keywords:	106	22.1 (18.6-26.1)	93	26.1 (21.8-30.8)	13	10.7 (6.3-17.4)	**<0.001**
	**-Weakness of the legs** Decreased ability to stand or walk including need to carry/lift the patient OR fallen OR found on the floor or corresponding place	93	19.4 (16.1-23.2)	81	22.7 (18.7-27.3)	12	9.8 (5.7-16.4)	**0.002**
	**-Fallen or being found on the floor** or corresponding place	59	12.3 (9.7-15.6)	57	16.0 (12.5-20.1)	2	1.6 (0.5-5.8)	**<0.001**
**11**	**History of deranged laboratory tests taken previous to EMS arrival** High blood sugar OR history of high CRP OR history of positive findings in blood culture taken previous to EMS arrival	43	9.0 (6.7-11.9)	30	8.4 (6.0-11.7)	13	10.7 (6.3-17.4)	0.452

**12**	**Abnormal neurology**<br>Focal neurological findings, photosensitivity, sound sensitivity, seizures or dysarthria	40	8.4 (6.2-11.2)	32	9.0 (6.4-12.4)	8	6.6 (3.4-12.4)	0.407
**13**	**Soiled patient**<br>Bloodstained or wetted from stool or urine	18	3.8 (2.4-5.9)	16	4.5 (2.8-7.2)	2	1.6 (0.5-5.8)	0.154
**14**	**Mood change**<br>Anxiety or fear OR feeling of depression	12	2.5 (1.4-4.3)	8	2.2 (1.1-4.4)	4	3.3 (1.3-8.1)	0.512 (F*)

EMS= Emergency Medical Services, CI=Confidence Interval, IV= Intravenous, CRP= C-Reactive Protein

*for comparison between EMS and non-EMS patients. P-values are presented without adjustment for multiple comparisons. In total 18 tests were performed. Bonferroni-adjusted significance level is 0.05/18=0.0028. P-values indicating significant differences after adjustment for multiple comparisons by Bonferroni correction are bolded and considered significant in the current study.

F* Fischer´s exact test was used for statistical analysis due to expected count being <5.

References:

1. Wallgren UM, Bohm KEM, Kurland L. Presentations of adult septic patients in the prehospital setting as recorded by emergency medical services: a mixed methods analysis. Scand J Trauma Resusc Emerg Med. 2017;25(1):23.
