# Supplementary material for: Keywords reflecting sepsis presentation based on mode of emergency department arrival: a retrospective cross-sectional study
Source: Int J Emerg Med. 2021 Dec 20;14:78. doi: 10.1186/s12245-021-00396-z (PMC8903703; doi:10.1186/s12245-021-00396-z)
Supplement: Supplementary file 4 — (PDF). Prevalence of keywords exceeding 20% based on mode of arrival and age group. A comparison of prevalence of all keywords* exceeding 20% among septic patients presenting to Södersjukhuset´s emergency department during 2013, based on mode of arrival AND age group. [file 12245_2021_396_MOESM4_ESM.pdf]

Additional file 4. Prevalence of keywords exceeding 20% based on mode of arrival and age group.

A comparison of prevalence of all keywords* exceeding 20% among septic patients presenting to the emergency department of Södersjukhuset during 2013, based on mode of arrival AND age group.

**Keyword***	**<65 yrs**			**65-74 yrs**			**≥75 yrs**		
	**EMS patients (n=69) Number (%)**	**non-EMS patients (n=68) Number (%)**	**P-value****	**EMS patients (n=76) Number (%)**	**non-EMS patients (n=26) Number (%)**	**P-value****	**EMS patients (n=212) Number (%)**	**non-EMS patients (n=28) Number (%)**	**P-value****
**Abnormal, or suspected abnormal temperature** In turn including primary keywords shivering OR hypothermia OR the following combined keywords: Confirmed or suspected fever, Confirmed abnormal temperature (confirmed fever or hypothermia)	45 (65.2)	52 (76.5)	0.147	54 (71.1)	18 (69.2)	0.860	133 (62.7)	17 (60.7)	0.835
**Pain** Abdominal, extremity, back, undefined, urinary tract, joint, chest, general, headache, throat, wound, painful muscle cramp, positive Pasternatsy´s sign (costovertebral angle tenderness)	32 (46.4)	55 (80.9)	**<0.001**	27 (35.5)	17 (65.4)	0.008	84 (39.6)	15 (53.6)	0.159
**Risk factors for sepsis** Known ongoing or recent infection, current antibiotic treatment, recent invasive procedures (see Additional file 2), substance abuse (see Additional file 2), compromised immune system (see Additional file 2), chronically compromised breathing (see Additional file 2)	40 (58.0)	34 (50.0)	0.349	25 (32.9)	18 (69.2)	**0.001**	45 (21.2)	10 (35.7)	0.086
**Temporal deterioration** Stated deterioration or expressions describing a temporal change	20 (29.0)	23 (33.8)	0.542	24 (31.6)	10 (38.5)	0.520	58 (27.4)	9 (32.1)	0.596
**Gastrointestinal symptoms** Vomiting, diarrhoea, reduced amount of stool, gastrointestinal bleeding, obstipation, pale faeces	24 (34.8)	20 (29.4)	0.501	18 (23.7)	9 (34.6)	0.275	62 (29.2)	4 (14.3)	0.096

**Abnormal skin**<br>Pale, wounds or wound infection, sweaty, cyanosis, redness, icterus, mottling, bruises, rash, blisters or peteckiae, change of skin turgor, exuding skin	25 (36.2)	21 (30.9)	0.507	18 (23.7)	2 (7.7)	0.076	53 (25.0)	6 (21.4)	0.680
**Abnormal urination**<br>Abnormal urination (such as hematuria without trauma, bad smelling or cloudy urine, increased frequency of urination) OR urinary tract pain OR decreased urinary volumes OR dysfunction of urinary catheters defined as obstruction/leakage/problematic urinary catheters including nefrostomias	11 (15.9)	12 (17.6)	0.789	24 (31.6)	4 (15.4)	0.110	57 (26.9)	10 (35.7)	0.328
**Abnormal circulation**<br>Weak pulse or difficulties to palpate the pulse, peripheral coldness, cardiac arrest, tachycardia, low blood pressure, prolonged capillary refill time or non-measurable circulatory variables	30 (43.5)	17 (25.0)	0.023	35 (46.1)	5 (19.2)	0.016	72 (34.0)	4 (14.3)	0.035
**Abnormal breathing**<br>Tachypnea, low oxygen saturation, airway secretions, breathing difficulties, cough, or obstructive breathing	32 (46.4)	12 (17.6)	**<0.001**	47 (61.8)	4 (15.4)	**<0.001**	106 (50.0)	9 (32.1)	0.075
**Loss of energy**<br>Defined as fatigue, weakness, faintness or similar expressions	17 (24.6)	11 (16.2)	0.290 (F*)	14 (18.4)	5 (19.2)	1.000 (F*)	60 (28.3)	6 (21.4)	0.508 (F*)
**Acute altered mental status**<br>Abnormal behaviour or level of consciousness (excluding previously known dementia or mental retardation without statement worse) OR abnormal verbal response defined as no/decreased verbal response .	25 (36.2)	3 (4.4)	**<0.001**	21 (27.6)	1 (3.8)	0.011	65 (30.7)	12 (42.9)	0.194
**Decreased mobility**<br>in turn including primary keywords remained sitting or lying in an abnormal way OR decreased miscellaneous mobility OR the following combined keywords: "weakness of the legs" and" fallen or being found on the floor".	16 (23.2)	2 (2.9)	**<0.001**	20 (26.3)	2 (7.7)	0.046	57 (26.9)	9 (32.1)	0.558

EMS= Emergency Medical Services. ED=Emergency Department.

*All keywords (combined or primary keywords) exceeding 20% prevalence in the entire sample of septic patients presenting to the ED.

**P-values are presented without adjustment for multiple comparisons. In total 13x3=39 tests were performed. Bonferroni-adjusted significance level is 0,05/39=0,0013. P-values indicating significant differences after adjustment for multiple comparisons by Bonferroni correction are bolded and considered significant in the current study.
