# Supplementary material for: Keywords reflecting sepsis presentation based on mode of emergency department arrival: a retrospective cross-sectional study
Source: Int J Emerg Med. 2021 Dec 20;14:78. doi: 10.1186/s12245-021-00396-z (PMC8903703; doi:10.1186/s12245-021-00396-z)
Supplement: Supplementary file 5 — (PDF). Prevalence of keywords exceeding 20% based on mode of arrival and gender. A comparison of prevalence of all keywords* exceeding 20% among septic patients presenting to Södersjukhuset´s emergency department during 2013, based on mode of arrival AND gender. [file 12245_2021_396_MOESM5_ESM.pdf]

Additional file 5. Prevalence of keywords exceeding 20% based on mode of arrival and gender.

A comparison of prevalence of all keywords* exceeding 20% among septic patients presenting to the emergency department of Södersjukhuset during 2013, based on mode of arrival AND gender.

Keyword*	Male			Female		
	**EMS patients (n=196) Number (%)**	**non-EMS patients (n=59) Number (%)**	**P-value****	**EMS patients (n=161) Number (%)**	**non-EMS patients (n=63) Number (%)**	**P-value****
**Abnormal, or suspected abnormal temperature** In turn including primary keywords shivering OR hypothermia OR the following combined keywords: Confirmed or suspected fever, Confirmed abnormal temperature (confirmed fever or hypothermia)	129 (65.8)	35 (59.3)	0.361	103 (64.0)	52 (82.5)	0.007
**Pain** Abdominal, extremity, back, undefined, urinary tract, joint, chest, general, headache, throat, wound, painful muscle cramp, positive Pasternatsy´s sign (costovertebral angle tenderness)	67 (34.2)	35 (59.3)	**0.001**	76 (47.2)	52 (82.5)	**<0.001**
**Risk factors for sepsis** Known ongoing or recent infection, current antibiotic treatment, recent invasive procedures (see Additional file 2), substance abuse (see see Additional file 2), compromised immune system (see see Additional file 2), chronically compromised breathing (see see Additional file 2)	65 (33.2)	35 (59.3)	**<0.001**	45 (28.0)	27 (42.9)	0.032
**Temporal deterioration** Stated deterioration or expressions describing a temporal change	57 (29.1)	18 (30.5)	0.833	45 (28.0)	24 (38.1)	0.139
**Gastrointestinal symptoms** Vomiting, diarrhoea, reduced amount of stool, gastrointestinal bleeding, obstipation, pale faeces	43 (21.9)	12 (20.3)	0.793	61 (37.9)	21 (33.3)	0.525
**Abnormal skin** Pale, wounds or wound infection, sweaty, cyanosis, redness, icterus, mottling, bruises, rash, blisters or peteckiae, change of skin turgor, exuding skin	52 (26.5)	14 (23.7)	0.667	44 (27.3)	15 (23.8)	0.591

**Abnormal urination**<br>Abnormal urination (such as hematuria without trauma, bad smelling or cloudy urine, increased frequency of urination) OR urinary tract pain OR decreased urinary volumes OR dysfunction of urinary catheters defined as obstruction/leakage/problematic urinary catheters including nefrostomias	61 (31.1)	11 (18.6)	0.062	31 (19.3)	15 (23.8)	0.448
**Abnormal circulation**<br>Weak pulse or difficulties to palpate the pulse, peripheral coldness, cardiac arrest, tachycardia, low blood pressure, prolonged capillary refill time or non-measurable circulatory variables	73 (37.2)	7 (11.9)	**<0.001**	64 (39.8)	19 (30.2)	0.181
**Abnormal breathing**<br>Tachypnea, low oxygen saturation, airway secretions, breathing difficulties, cough, or obstructive breathing	98 (50.0)	8 (13.6)	**<0.001**	87 (54.0)	17 (27.0)	**<0.001**
**Loss of energy**<br>Defined as fatigue, weakness, faintness, or similar expressions	43 (21.9)	11 (18.6)	0.587	48 (29.8)	11 (17.5)	0.059
**Acute altered mental status**<br>Abnormal behaviour or level of consciousness (excluding previously known dementia or mental retardation without statement worse) OR abnormal verbal response defined as no/decreased verbal response .	63 (32.1)	6 (10.2)	**0.001**	48 (29.8)	10 (15.9)	0.032
**Decreased mobility**<br>in turn including primary keywords remained sitting or lying in an abnormal way OR decreased miscellaneous mobility OR the following combined keywords: "weakness of the legs" and" fallen or being found on the floor".	56 (28.6)	9 (15.3)	0.040	37 (23.0)	4 (6.3)	0.004

EMS=Emergency Medical Services. ED=Emergency Department.

*All keywords (combined or primary keywords) exceeding 20% prevalence in the entire sample of septic patients presenting to the ED.

**P-values are presented without adjustment for multiple comparisons. In total 26 tests were performed. Bonferroni-adjusted significance level is 0,05/26=0,0019. P-values indicating significant differences after adjustment for multiple comparisons by Bonferroni correction are bolded and considered significant in the current study.
