# Supplementary material for: Keywords reflecting sepsis presentation based on mode of emergency department arrival: a retrospective cross-sectional study
Source: Int J Emerg Med. 2021 Dec 20;14:78. doi: 10.1186/s12245-021-00396-z (PMC8903703; doi:10.1186/s12245-021-00396-z)
Supplement: Supplementary file 6 — (PDF). Prevalence of keywords exceeding 20% based on mode of arrival and sepsis severity. A comparison of prevalence of all keywords* exceeding 20% among septic patients presenting to Södersjukhuset´s emergency department during 2013, based on mode of arrival AND sepsis severity. [file 12245_2021_396_MOESM6_ESM.pdf]

Additional file 6. Prevalence of keywords exceeding 20% based on mode of arrival and sepsis severity.

A comparison of prevalence of all keywords* exceeding 20% among septic patients presenting to the emergency department of Södersjukhuset during 2013, based on mode of arrival AND sepsis severity.

**Keyword***	**Severe sepsis**			**Non-severe sepsis**		
	**EMS patients (n=218)**	**non-EMS patients (n=30)**	**P-value****	**EMS patients (n=128)**	**non-EMS patients (n=83)**	**P-value****
**Abnormal, or suspected abnormal temperature** In turn including primary keywords shivering OR hypothermia OR the following combined keywords: Confirmed or suspected fever, Confirmed abnormal temperature (confirmed fever or hypothermia)	138 (63.3)	23 (76.7)	0.150	89 (69.5)	59 (71.1)	0.810
**Pain** Abdominal, extremity, back, undefined, urinary tract, joint, chest, general, headache, throat, wound, painful muscle cramp, positive Pasternatsy´s sign (costovertebral angle tenderness)	75 (34.4)	21 (70.0)	**<0.001**	61 (47.7)	61 (73.5)	**<0.001**
**Risk factors for sepsis** Known ongoing or recent infection, current antibiotic treatment, recent invasive procedures (see Additional file 2), substance abuse (see Additional file 2), compromised immune system (see Additional file 2), chronically compromised breathing (see Additional file 2)	59 (27.1)	11 (36.7)	0.273	49 (38.3)	42 (50.6)	0.078
**Temporal deterioration** Stated deterioration or expressions describing a temporal change	67 (30.7)	7 (23.3)	0.406	31 (24.2)	32 (38.6)	0.026
**Gastrointestinal symptoms** Vomiting, diarrhoea, reduced amount of stool, gastrointestinal bleeding, obstipation, pale faeces	54 (24.8)	10 (33.3)	0.315	47 (36.8)	20 (24.1)	0.054

**Abnormal skin**<br>Pale, wounds or wound infection, sweaty, cyanosis, redness, icterus, mottling, bruises, rash, blisters or peteckiae, change of skin turgor, exuding skin	57 (26.1)	6 (20.0)	0.468	37 (28.9)	21 (25.3)	0.567
**Abnormal urination**<br>Abnormal urination (such as hematuria without trauma, bad smelling or cloudy urine, increased frequency of urination) OR urinary tract pain OR decreased urinary volumes OR dysfunction of urinary catheters defined as obstruction/leakage/problematic urinary catheters including nefrostomias	57 (26.1)	7 (23.3)	0.741	33 (25.8)	19 (22.9)	0.634
**Abnormal circulation**<br>Weak pulse or difficulties to palpate the pulse, peripheral coldness, cardiac arrest, tachycardia, low blood pressure, prolonged capillary refill time or non-measurable circulatory variables	115 (52.8)	15 (50.0)	0.777	19 (14.8)	10 (12.0)	0.565
**Abnormal breathing**<br>Tachypnea, low oxygen saturation, airway secretions, breathing difficulties, cough, or obstructive breathing	127 (58.3)	8 (26.7)	**0.001**	53 (41.4)	16 (19.3)	**0.001**
**Loss of energy**<br>Defined as fatigue, weakness, faintness or similar expressions	58 (26.6)	7 (23.3)	0.702	30 (23.4)	14 (16.9)	0.251
**Acute altered mental status**<br>Abnormal behaviour or level of consciousness (excluding previously known dementia or mental retardation without statement worse) OR abnormal verbal response defined as no/decreased verbal response	111 (50.9)	16 (53.3)	0.804	0 (0.0)	0 (0.0)	-
**Decreased mobility**<br>in turn including primary keywords remained sitting or lying in an abnormal way OR decreased miscellaneous mobility OR the following combined keywords: “weakness of the legs” and” fallen or being found on the floor”	62 (28.4)	3 (10.0)	0.031	27 (21.1)	9 (10.8)	0.053

EMS= Emergency Medical Services. ED= Emergency Department.

*All keywords (combined or primary keywords) exceeding 20% prevalence in the entire sample of septic patients presenting to the ED.

**P-values are presented without adjustment for multiple comparisons. In total 26 tests were performed. Bonferroni-adjusted significance level is 0,05/26=0,0019. P-values indicating significant differences after adjustment for multiple comparisons by Bonferroni correction are bolded and considered significant in the current study.
